# Atlanta Academy of Medicine

**Published:** 1872-02

**Authors:** J. T. Johnson

**Affiliations:** Reporter


### ATLANTA ACADEMY OF MEDICINE.

J. T. JOHNSON, M.D., REPORTER.

DECEMBER 11, 1871.

The President, Dr. Logan, in the chair.

Dr. W. H. Cumming read an interesting article—an extract from the proceedings of the Canadian Institute, of Toronto—descriptive of the process used by Dr. Oldwright for drawing off the pus in empyema, without the admission of air into the chest cavity. The paper is here given in full:

"The regular meeting of the medical section of the Canadian Institute was held last evening in the rooms on Richmond street east, when Dr. Oldwright introduced a very interesting case of empyema, treated by the use of the syphon tube. The subject was a boy four years and nine months old, who, in December last, was attacked with acute bronchitis. After three or four weeks of treatment, a dullness and bulging was observed on the right side of the chest; a week afterward, a dullness was observable on the left side of the chest, and it became apparent that the cavity of the chest was distended with fluid. On the operation of tapping being performed, about six ounces of pus came away, which was drawn off under water. A week later, the tapping was repeated—this time between the ninth and tenth ribs, in the back portion of the chest, and the pus was again drawn off by means of a syringe and elastic tubing. The parents at one time were averse to the mode of treatment pursued, believing that it caused pain to the child; but Dr. Oldwright at length persuaded them to allow him to use the syphon process. The operation consists in the introduction of a large trocar and canula into the side, between the fifth and sixth ribs; on withdrawing the trocar, a tube filled with water was introduced into the chest, and the canula slipped off over the tube, one end of which was then in the chest, and the other extremity placed in a glass of water, drawing from the cavity the pus accumulated therein. The glass was then refilled with clean lukewarm water, the lower end of the tube being kept closed during its removal; and on raising the glass, the water flowed into the chest. The process of filling and emptying the chest was repeated until the discharge was quite clear, and then the process was repeated with a weak solution of carbolic acid, the end of the tube being afterward tied to prevent the destruction of the action of the syphon; the end was coiled up on the chest, and kept in its place by strips of plaster. The discharge in May last was about four ounces daily, but now it is only very slight—about a tea spoonful—and the patient has so far recovered as to be able to take part in the sports of his playmates. By means of a bottle, into the neck of which the end of the tube was placed, the Doctor showed that all the fluid that could be got into the cavity of the chest was about a tea-spoonful, and the tube will soon be forced out by the closing of the cavity."

Dr. Cumming added, that the interesting point was the flowing of the fluid both ways—first externally, and then, by raising the basin, internally, washing out the cavity of the chest—all accomplished without the introduction of any air into the cavity. The tube must be a small one, about the size of the trocar, as the canula must be slipped over it. After the operation is concluded, the tube is secured to the chest by adhesive plaster, till it is necessary to bring it again into requisition.

Dr. Harden.—Why does the air not enter the chest by the side of the tube?

Dr. Cumming.—Because it is pressed tight by the parietes of the chest. If it were necessary—though it is not—this could be further secured by some adhesive substance.

Dr. W. F. Westmoreland.—Must regard this a great improvement over the ordinary plan. Has it been suggested before?

Dr. Cumming.—Never heard of it.

Dr. Westmoreland.—The admission of air within the chest is always an unfortunate accident, even if there follow no change in its contents. The air often gives such pain as to call for the use of immediate stimulants and restoratives.

Dr. John Stainback Wilson.—No matter whether the idea was suggested before or not; it is an exceedingly good one.

Thinks he read the suggestion several years ago, in some medical journal. The details may not have been precisely the same as in the present instance, but the idea of the exclusion of the air by means of the syphon was the same. It is certainly a good plan, and this gentleman deserves credit for carrying it out. Himself treated a case of empyema several years ago, in which the use of this apparatus would doubtless have saved much pain, though the patient finally recovered. The effusion was very great, displacing the heart, and even pushing it to the right of the sternum. There was an opening into one of the bronchi, in addition to the one made through the chest-walls. Pus was thus discharged with the expectoration. Air also escaped, with considerable noise, through the external opening, as the fluid flowed out.

Dr. J. G. Westmoreland desires to report a case, interesting to him not because the symptom is unusual—for we have all treated it—but because it is annoying and obstinate one; and because, further, he would like to hear from the members concerning their experience with it. It is a case of persistent nausea attending the first month or two of pregnancy. It has been almost constant, day and night, for about six weeks. Was doubtful at first whether it was pregnancy or a disease of the womb, as there are some symptoms indicative of the latter. The vagina is exceedingly sensitive, rendering digital examination very painful. She was with difficulty prevailed upon to allow the use of the speculum, for dread of the pain. The womb presented a natural appearance; was thus convinced that it was pregnancy. Although the nausea is so distressing, there are no symptoms of danger. She is a stout, healthy woman, in good condition. Has used successfully, in some cases, the oxalate of cerium; but this and other usual remedies were of no avail in this instance.

Dr. W. H. Cumming.—From the statement of Dr. Westmoreland, should regard the hyperæsthesia as the peculiarity of this case—as a complication, in fact. Should try some local remedies, looking, if this trouble should be relieved, for an improvement in the nausea.

Dr. J. G. Westmoreland.—Would add, that there are pains about the womb, in the back, and on pressure over the womb,

There is also leucorrhœa. No fever, no displacement, no vaginitis. The tenderness has been benefitted somewhat by washes, etc.

Dr. W. M. Wells.—Is there a possibility of gonorrhœa?

Dr. Westmoreland.—None.

Dr. Bozeman.—Does the nausea come on regularly in the morning?

Dr. Westmoreland.—It lasts through the day, and night also.

Dr. John Stainback Wilson.—Is a strong advocate of the theory of pregnancy and parturition being a natural process. Does not believe that the diseases of pregnancy, as they are commonly called, are necessary concomitants of that state. Believes that if the woman would live right, observing rigidly the laws of health, she would escape these diseases—the vomiting with the others. Would suggest local means in this case, especially water by means of the vaginal irrigator, using it first slightly warm, and gradually making it colder.

Dr. J. G. Westmoreland.—Would reply to Dr. Wilson, that there is no danger yet of the natural process of parturition in this case. The sensitiveness existed in some degree before pregnancy; seems naturally so.

Dr. W. F. Westmoreland.—Would like to hear from the members, who have paid particular attention to the diseases of women, their opinion as to what produces the nausea of pregnancy. And why are some so affected, while others escape?

Dr. Wilson.—As the question seems directed particularly to me, will answer, that it is engendered by bad habits; it is a morbid irritability of the system. If the patient will live right, it can be avoided nine times out of ten. Has no doubt of the possibility of its prevention.

Dr. J. G. Westmoreland.—This is a very stout, healthy woman. Looks as if she had never been sick.

Dr. Cumming.—Is it the first pregnancy?

Dr. Westmoreland.—Has had several pregnancies. Usually experiences the ordinary sickness.

Dr. Logan.—Is the womb lower than natural?

Dr. Westmoreland.—Not at all.

Dr. E. L. Connally stated that he used, in one case of persistent nausea, half a drachm of extract belladonna, applied for several mornings to the os tincæ. The woman was able to be on the street in a week. This was the fifth pregnancy. In previous ones, she had been confined to bed for six months. The idea was obtained from the writings of Cazeaux.

Dr. Westmorelond.—That is short, and to the point. Will try it.

Dr. Bozeman.—Is inclined to attribute the nausea to some general nervous irregularity. Would try tonics, as quinine, strychnia, etc. The diet should also receive careful attention. Has often known the application of chloroform over the stomach to relieve it. Has also known a tight bandage to succeed, as it sometimes does in sea-sickness.

The President called on Dr. Speer, of Pittsburg, Pennsylvania—present with the Academy—for his experience with the disease.

Dr. Speer stated, in reply, that his observation had furnished nothing new or interesting. It had occurred to him, during this discusion, that he would be disposed to try the application of carbonic acid gas to the vagina. Chemistry now furnishes many modes of producing the gas. Read, in a recent number of a medical journal, of an apparatus for producing it, similar to that for generating nitrous oxide for inhalation.

Dr. Baird reports that he has recently had under his care a case of scarlet fever, of a malignant type. The patient was a girl, aged nine years. She retired as usual at nine o'clock, but awoke at two with a rigor. When he first saw her, at twelve the next day, she was insensible. Vomiting had been constant during the night; pulse one hundred and ten; temperature not excessive. Saw her again in the afternoon. Vomiting continued; there was now a slight eruption. Saw her the next morning. Had had many convulsions. Again saw her in the afternoon. There was active delirium; no rest, though opiates had been given; the eruption well-marked. She died just forty-eight hours from the time of attack. Dr. Logan (who has been called off) had mentioned to himself three cases that occurred in his practice.

Dr. Cumming.—It seems that it would be well, if we are to have an epidemic of this disease, that we should attempt to limit the contagion. In England, they seem to be of opinion that it is not necessary to have the second case, after one is attacked. They accomplish this by insolation and by the use of carbolic acid—sponging with it, and hanging over the door a blanket saturated with it; also, rigorously destroying all clothing used about the patient. It would seem that the desquamating scales in the convalescing stage are capable of communicating the disease. We should use any amount of energy to prevent its spread.

Dr. W. F. Westmoreland.—We would like to hear Dr. Cumming's mode of preventing its spread. Here are three or four cases reported as occurring in different parts of the city. It may appear spontaneously, without any direct communication. It would be difficult to correct the atmosphere, but it must be done to arrest an epidemic.

Dr. Harden then related some facts occurring under his observation, showing its undoubted communicability by being carried many miles in the clothing, the person so carrying it not necessarily becoming diseased.

Dr. Baird confirmed Dr. Harden's views by saying that he had himself had the disease, and took it in the way described.

Dr. W. F. Westmoreland thought that, while it is contagious, it may arise without having its origin from another patient.

A number of the members joined in the discussion of its contagious or infectious character.

Dr. John Stainback Wilson, upon invitation, read an essay to prove that parturition was naturally a painless process, and is rendered otherwise only by degenerated habits of living.

At its close, the essay was discussed by Drs. Cumming, Westmoreland, Wells and others, all taking grounds against the views of Dr. Wilson.

December 18th, 1871.

The President, Dr. Logan, in the chair.

Dr. J. G. Westmoreland, having been asked for the progress of his case of persistent nausea attending pregnancy, states that he has not been able to thoroughly test the action of extract of belladonna, locally applied, owing to the extreme sensitiveness of the vagina rendering its application difficult. Will make further endeavors. The bandage around the body he has tried, but no benefit resulted.

Dr. W. H. Cumming.—Has seen some reports of the use of wine of ipecac, given in doses of one drop every hour, with admirable effect.

Dr. W. P. Harden.—Would suggest hot water—not merely warm, as it is often used to promote vomiting, but as hot as can be borne. Has tested this in several instances of vomiting, though not in the nausea of pregnancy, and found it to answer the purpose finely.

Dr. Speer, of Pennsylvania, by request of the President, made some further statements (he having alluded to it at the previous meeting) of the use of carbonic acid gas, locally applied, in painful affections of the vagina and womb. He read some extracts from an article in the February (1871) number of the Medical and Surgical Reporter, of Philadelphia, showing its successful use by a number of eminent physicians. The writer of the article—Dr. Th. A. Demme, of Philadelphia—detailed several cases of his own experience with the remedy, as used in parturition. In these cases, the pain was alleviated to a remarkable degree—in some of them, indeed, abolished. Nor was its effect less marked in quickly dilating the *os uteri*, which, by its rigidity, had retarded the labor for many hours.

The materials used by Dr. Demme for making the gas were about one-half an ounce each of bicarbonate of soda and tartaric acid. These were mixed in water in a common pint bottle, and the gas conveyed to the vagina and womb through a rubber tube about three feet in length. He afterward had constructed, as more elegant and convenient, a rubber bag, with two openings—one for the attachment of the tube, the other for the introduction of the materials.

Dr. Demme also referred to the use of this remedy in painful uterine affections—especially chronic and malignant ones—by Dr. Dewees, of Philadelphia, Prof. Majon, of Geneva, Dr. Rossi, of Italy, (this case given by Dr. Pareira, in the second edition of his work on Materia Medica), the late Prof. Simpson, of Edinburgh, and Prof. Scanzoni, of Vienna, who used it extensively. These are all reported as succeeding remarkably in thus relieving pain. Dr. Dewees, in 1835, in his "Treatise on the Diseases of Females," refers to his successful use of the gas. He obtained the gas by diluted sulphuric acid and carbonate of lime.

Dr. Westmoreland states that when he was in Paris, in 1853–'54, the practice was common of applying this gas as an anæsthetic to most, or many, of the mucous membranes. On a second visit, in 1856, it was still more used; was often applied even to the bladder. Its use was principally confined to the relief of pain in incurable cases, and with the happiest effect. He himself often used it here in Atlanta in such cases. Remembers to have thus relieved one case of intense hyperæsthesia of the vagina. There is no difficulty in making the gas. Used a bladder, or retort, provided with a stop cock. A French writer taught that poultices exerted their good effect through the carbonic acid gas generated from the materials used. There is no doubt but this agent is anæsthetic to mucous membranes. Has never seen it tried in parturition; would expect benefit from it if there were an irritable vagina or os.

Dr. J. G. Westmoreland desires to know if there would be a possibility of permanent benefit in the nausea of his case, or would it be merely temporary?

Dr. Speer believes there would be more permanency than in most local anæsthetics; at any rate, it can be often repeated, if necessary. He cannot speak from his own experience with the gas.

Dr. Cumming thinks its use might be advisable, in order to render the vagina tolerant of the application of the belladonna.

Inquiry was made if any more cases of scarlet fever had come under the observation of the members.

Dr. Logan stated that he had only met with three cases. (These are the same referred to by Dr. Baird, at the last meeting of the Academy.—Reporter.) These all occurred in the same family. None were malignant, though all were wellmarked. Has heard of but one malignant case, that of Dr. Baird. The appearance of his cases was singular, as there were no other cases in the neighborhood, making it difficult to account for the origin. Heard only an indefinite rumor of other cases in the city, but the rumor was probably incorrect. One or two ladies of the family had recently returned from a town of upper Georgia where the disease was prevailing. It may have possibly been communicated in this way. The coincidence, to say the least, was a remarkable one.

Dr. Baird stated that he had met with no other case. Several members of the family of his first patient had suffered from sore throat.

Dr. Cumming.—Since we are on eruptive fevers, may mention that he has heard from Dr. Etheridge, legislator, that the Swedes have brought small-pox into Putnam county.

Dr. Vaughan, of Arkansas—present at the meeting—was called upon for something of interest from his portion of the country. Stated that the chief diseases met in his practice were malarious. Was asked by Dr. Wells if he had met any malarious hæmaturia. Replied, that he had met a malignant form of malaria of a hemorrhagic tendency. Till recently, their treatment of it was a failure; now, they have arrived at something more rational and satisfactory. There seems, in such cases, to be a perfect sedation—an overwhelming of the system; all the functions of the body are arrested. The bile is not excreted, and adds further to the poisoned condition. The treatment is to arrest the paroxysm of depression, and support the system by brandy, quinine, etc. The more general impression is that quinine should be given in small doses; that large ones aggravate it. Should avoid the use of anything that arrests the secretions. Sulphur is highly regarded by some as a purgative in this affection.

Dr. S. H. Stout.—Has now under treatment two cases from Arkansas. There is no hæmaturia, but the inroads on the system are lamentable. The patients are a young gentleman and lady, aged respectively eighteen and sixteen. The young man is much emaciated; large ague cake; has had a large ulcer on his leg for several years; first had a passive hemorrhage under the skin, and then followed ulceration. The young lady, in addition to other symptoms, as ague cake, anæmia, etc., has large spots of purpura hemorrhagica over the surface. There is also a disposition to œdema. Put them upon the use of arsenic; also quinine, myrrh, sulphate of zinc, and iron. There is a little improvement. Some of the spots disappear, but are succeeded by others.

This malarious hæmaturia seems to be on the increase. As people dwell long in these districts, the succeeding generations seem to be more frail—the constitution becomes impaired. Dr. Vaughan's idea, of small doses of quinine being more beneficial in these cases, has truth in it; large doses are sedative, and may interfere with the functions of the stomach. He never used the sulphur, but it strikes him as a good remedy. Sulphurous acid is used with fine effect in zymotic diseases. The hyposulphites sometimes cut short an attack of small-pox. It is probable that arsenic, so often used in malarious poisoning, is not really an anti-periodic, but that it destroys the germ of the disease. It may not be out of place to remark here, that, according to his observation, we have an increase of malaria in Atlanta every year.

To return to the cases he has mentioned; neither of the patients has been affected with hæmaturia, but believes that the young lady would so suffer were it not for the determination to the surface, as indicated by the purpura.

Dr. Vaughan.—Has himself been struck with the fact, that he has never seen the purpura and the hæmaturia present in the same case; but always, if one was present, the other was wanting.

Dr. Stout.—Any one, who has passed through epidemics, has noticed that there is a tendency in each to disease, or complication, of certain organs. Each epidemic of scarlet fever presents a tendency to complication of different organs. This hæmaturia is nothing but old-fashioned malaria, with this peculiar direction of the poison.

Dr. Raushenberg.—I would like for Dr. Vaughan to detail the symptoms of this disease.

Dr. Vaughan.—Primarily, there is an extraordinary depression, ushered in by a chill; this followed directly by the bloody urine. The condition is asthenic; there is an acceleration of the pulse, but no increase of its volume. There is but little, if any, reaction; occasionally, there is slight periodicity. A great many of those attacked die; many live from three to five days, but sometimes not more than twenty-four hours. On entering the room of such a patient, one cannot fail to be struck with his peculiar skin and general appearance—very similar to that of a person attacked with yellow fever.

Dr. Raushenberg.—It seems to me that this is a malignant form of malarial fever.

Dr. W. F. Westmoreland.—If so, why is not the quinine treatment applicable to it?

Dr. Wells.—In answer to that question, will give some of his experience with the affection. Treated a number of cases of it, in Alabama, several years ago. Has no doubt but that it is strictly malarious in its origin, but it hardly deserves the name of fever. So far as his knowledge went, but few negroes were attacked. In every instance, the attack was ushered in by a chill; often, the patient had been subject to chills beforehand. Various plans of treatment were adopted. Some relied on hyposulphite of lime; some, on tincture of iron; some, on strychnia, to stay the paroxysm. Adopted the latter himself in 1868, in conjunction with hyposulphite of lime. Gave quinia in one case, that of a child. The attack had just commenced, but he was convinced, in two hours, that the remedy had done harm; the child died in four hours, or less. Quinia is not eliminated from the kidneys. Dr. Keenan, in one case, gave tannic acid; it had the desired effect on the urine, but yet the man died. Thinks the judicious use of it advisable—that it would arrest the blood, but not the urine.

In 1868, he had nine cases; two of these died. In one of the nine, blisters were used; they were followed by sloughing, and oozing from the surface. There was black vomit in one case—similar, he thinks, to that of yellow fever. Yellowness of the skin was always present, coming on with the chill. He also observed hemorrhage from the bowels supervening later in the disease. Quinine fails to arrest the attack; is opposed to it, and to opiates even in small doses. Belladonna seemed to prove favorable in one case, keeping the skin hot, while it was clammy cold in most of them.

Dr. Raushenberg.—Are hale and hearty persons ever attacked?

Dr. Wells.—I think they occasionally are; but generally the patients were previously in ill-health, suffering, perhaps, from intermittent fever.

Dr. Harden.—This disease, as he understands it from the description, seems similar to the bloody murrain in cattle, which is itself characterized by bloody urine.

Dr. W. H. Cumming.—Inquires if there have been any instances of strong, hearty men being struck down, as it were, suddenly. We do not, and never have, any of us, lived in a really malarious district, where the poison exerts all its concentrated power. Has seen, in China, two hale, hearty British soldiers overcome almost instantly by the power of malaria. It didn't break out in your petty fiddlings, like chills, but the intensity literally crushed the man at one blow.

There were three sets of troops under his observation—the white, the Hindoo, and a mixed race. The contrast between the Caucassian and the Hindoo was remarkable. Many of the Europeans died in a few hours. Out of a regiment of three hundred and sixty, sixty died in six weeks, and many more subsequently. There was not an instance of this intense poisoning among the Hindoos. Though nearly all of them were sick, they recovered rapidly; but when one of the Europeans was attacked, his condition was such that you felt he was bound to die—that any power great enough to restore him could make a new man.

